# Conservation Genetics of the Loggerhead Sea Turtle, *Caretta caretta*, from the Central Mediterranean: An Insight into the Species’ Reproductive Behaviour in Maltese Waters

**DOI:** 10.3390/ani14010137

**Published:** 2023-12-30

**Authors:** Adriana Vella, Noel Vella

**Affiliations:** Conservation Biology Research Group, Department of Biology, Faculty of Science, University of Malta, MSD 2080 Msida, Malta

**Keywords:** maternity test, paternity test, mtDNA control region, microsatellites, philopatry, polyandry, multiple paternity, renesting

## Abstract

**Simple Summary:**

This work represents the first scientific study using genetic markers to better understand the reproductive behaviour of the loggerhead sea turtle from the Central Mediterranean through dead hatchlings and undeveloped dead embryos collected from recorded nesting sites between 2020 and 2022. Data generated from the genetically analysed specimens were used for parentage analyses. Results of this research show that some turtles laid more than one nest within the same nesting season while we also detected an instance of multiple paternity. These findings contribute to a better understanding of the biology of the species in Maltese waters, which is a requirement for effective conservation management.

**Abstract:**

Loggerhead sea turtle, *Caretta caretta* (Linnaeus, 1758), nestlings were investigated through specimens found dead either after hatching or unhatched (*n* = 120) from eight nests around the Maltese islands (Central Mediterranean). Molecular genetics was used to conduct maternity and paternity tests of the collected specimens utilizing expanded mitochondrial DNA sequences from the control region (858 bp) and 25 microsatellite loci (12 dinucleotide loci and 13 tetranucleotide loci). Mitochondrial data produced two haplotypes, CC-A2.1 and CC-A3.1, with the most common haplotype being present in seven nests. Microsatellite data revealed the identity of six different females that were involved in the deposition of the eggs in the eight turtle nests analysed. This confirms that two females laid multiple nests. Additionally, microsatellite data allowed for the determination of multiple paternity, with one clutch being sired by two fathers. These results are useful for monitoring the genetic diversity of loggerhead sea turtle nestlings and of the turtle mothers and fathers contributing to future turtle offspring, which rely on Maltese sandy beaches for their successful start to life. Effective conservation management benefits from merging scientific knowledge with effective measures at potential nesting sites to avoid losses of nestlings caused by human negligence.

## 1. Introduction

The Mediterranean Sea hosts the green sea turtle *Chelonia mydas* (Linnaeus, 1758) and the loggerhead sea turtle *Caretta caretta* (Linnaeus, 1758) [1,2,3], both of which are known to nest in the region [2,4]. *Chelonia mydas* nesting sites are mostly restricted to the Eastern Mediterranean [2,5] while the major nesting sites for *C. caretta* were historically also associated with the Eastern Mediterranean; however, in the last few decades, the geographical centre for Mediterranean *C. caretta* nesting sites shifted towards the Central Mediterranean due to increasing records in the Western Mediterranean [2,6,7]. The Mediterranean Sea is also frequented by leatherback turtles, *Dermochelys coriacea* (Vandelli, 1761), migrating occasionally from the Atlantic Ocean [3,8,9]; however, there are no nest records for this species in the region.

According to the IUCN, the loggerhead sea turtle is listed as vulnerable at a global level [10] and as least concern at the Mediterranean subpopulation level [11]. It has also been enlisted in a number of international frameworks, including the Convention for the Protection of the Mediterranean Sea against Pollution (Barcelona Convention—Annex II) [12], the Convention on Migratory Species (CMS—Appendix I) [13], the Convention on the Conservation of European Wildlife and Natural Habitats (Bern Convention—Appendix II) [14], the Convention on International Trade in Endangered Species (CITES—Appendix I) [15], and the Protocol of the Barcelona Convention concerning Specially Protected Areas and Biological Diversity in the Mediterranean (SPA/BD—Annex II) [16]. Moreover, the International Commission for the Conservation of Atlantic Tunas (ICCAT) and the General Fisheries Commission for the Mediterranean (GFCM) provide recommendations to fishery managers in relation to the incidental bycatch of sea turtles in the Mediterranean Sea [17,18] while the Marine Strategy Framework Directive within the Biodiversity Descriptor allows for good environmental status assessments of sea turtles [19].

Globally, the loggerhead sea turtle is split into a number of regional management units (RMUs), which have been identified through mitochondrial DNA (mtDNA) and nuclear DNA (nDNA) [3,20]. This structure is mostly based on nesting populations due to female philopatry, exhibiting nesting site fidelity. Nonetheless, population structures are more complex due to the male-mediated gene flow and overlapping populations, especially during migration. Mitochondrial DNA control region (mtDNA CR) data for the Mediterranean Sea indicate that *Caretta caretta* populations have colonized the Mediterranean Sea during the Pleistocene [21], have survived the glacial periods in warm refugia within the south-eastern parts of the Mediterranean Sea [21], and have undergone multiple colonization events [20]. Additionally, the mtDNA CR also exhibits enough variation and structure at a small scale that the RMU of the Mediterranean Sea can be split into smaller management units [2,21,22], which may be necessary for the management of rookeries.

The species is prone to various anthropogenic pressures. The effects of climate change, including rising sea levels, changes in tides, waves, precipitation patterns, and changing temperatures, impose threats to the development of sea turtle embryos [23,24,25,26] while rising sand temperatures skew the primary sex ratios towards females, leading to the potential feminization of several sea turtle populations while lowering the hatching success and hatchling fitness, including those of *C. caretta* [20,27,28]. These climatic changes together with other human-induced threats, such as coastal development, disturbances, and vessel traffic, also affect the behaviours of adults, including their approach to land [24,29]. Therefore, there is an increasing requirement for more scientific data collection on the reproductive biology of sea turtles to design conservation management strategies that improve the resilience and survival of these species.

Therefore human activities impact both the nestlings and free-swimming sea turtles through fast-changing and deteriorating marine environments with greater pollution, disturbances, and injuries caused by boat propellers and fishing gear [1,2,30,31,32,33,34]. The Mediterranean Sea is highly exposed to such anthropogenic activities [35], with most of them increasing in frequency during late spring and summer, which coincides with breeding migration and the mating period of *C. caretta*, which peaks between April and May [2]. Therefore, the cumulative effect of these threats would result in an increased mortality rate and smaller population sizes, reducing reproductive fitness while disturbing the natural behaviours of this species. Consequently, anthropogenic effects on the nesting sites, nests, nesting behaviours, mating behaviours, and migratory patterns influence the reproductive potential of this species.

The objectives of this first scientific research paper on loggerhead sea turtle nestlings from the Maltese islands are to (1) understand the genetic relationship between different nests and whether the females return to nest within the same season (renesting) and site fidelity and (2) estimate the frequency of multiple paternity. Here, mtDNA sequences and nuclear DNA genotyping were used as tools to allow for the computational sibship and parentage investigation [36,37] of all the recorded nests between 2020 and 2022.

## 2. Materials and Methods

### 2.1. Study Area and Background Information

**Figure 1:**
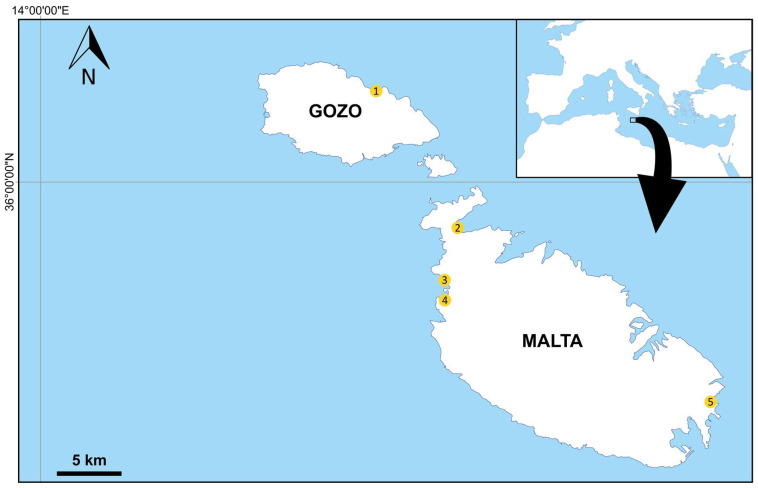
Map indicating the five nesting beaches recorded on the Maltese islands between 2012 and 2023. 1. Ramla Bay; 2. Għadira Bay; 3. Golden Bay; 4. Ġnejna Bay; 5. Fajtata Bay.

This study focuses on *C. caretta* nests from the Maltese archipelago. This archipelago is situated in the Central Mediterranean, approximately 95 km south of Sicily and 285 km from the Tunisian coast (Figure 1), and is characterized by two main islands, Malta and Gozo. In recent years there have been thirteen reported nests, one each in 2012, 2016, and 2018; six in 2020; one each in 2021 and 2022; and an additional two in 2023 (Table 1).

We looked into the remains from nests laid between 2018 and 2022. Given that the 2018 nest did not contain any dead individuals then, the genetic analyses focused on eight nests laid between summer 2020 and 2022, representing four beaches around the Maltese islands (Figure 1). For six of the nests analysed, the local Environment and Resource Authority (ERA, *pers. comm*.) was alerted about the nests when the respective mothers laid their eggs and the nests were monitored until the juveniles hatched. In one of these cases, the eggs were dug up and transferred to an incubator, given that the integrity of the nest was assessed as being compromised by adverse environmental conditions (ERA, *pers. comm.*). In two instances, the nest was unknown prior to the discovery of the hatchlings.

Locally, the species is strictly protected under *Flora, Fauna, and Natural Habitats protection regulations* [38] and a number of marine Natura 2000 sites have been designed to further protect the species, including MT0000113; MT0000115; and MT0000116 [39]. Consequently, any dead hatched and dead unhatched specimens were collected from the respective nests by the local authority ERA; then, they were handed on for tissue sampling and scientific investigation by AV in accordance with handling and research ERA permits.

### 2.2. Sample Collection, DNA Extraction, PCR Amplification, and Sequencing

Tissue samples from dead unhatched individuals or dead hatchlings were excised and stored in 100% ethanol. The total genomic DNA was then extracted from tissue samples using the GF-1 Tissue DNA Extraction Kit (Vivantis Technologies, Shah Alam, Malaysia) following the manufacturer’s manual. The concentration of the purified DNA was estimated using Qubit (ThermoFisher Scientific, Waltham, MA, USA).

Given that all samples within a clutch come from the same mother, for mtDNA, analyses of two specimens per nest were randomly chosen, except for the nest CRA, where only one individual was available and, thus, the same individual was analysed twice. For the selected specimens, the mtDNA CR was amplified using LCM15382 and H950 [40] following the work of Shamblin et al. [41]. The PCR products were then purified and sequenced with their respective forward and reverse primers via the ABI3730XL sequencer (Applied Biosystems, Waltham, MA, USA).

Twelve dinucleotide microsatellite loci [42,43,44,45,46] and thirteen tetranucleotide microsatellite loci [47,48] were selected for more detailed maternal and paternal analyses of each nest. Each sampled specimen was analysed for the 25 microsatellites (Table 1) that were all tagged by M13 tails; fluorescently labelled using 6-FAM, VIC, NED, or PET; and amplified following published temperature profiles [46,47,48] (Supplementary Table S1). PCR products were size-scored through Applied Biosystems ABI3730XL, using Liz600 as the fluorescent size standard. During these analyses, 12 specimens, representing 10% of the sample size, were randomly chosen and were run twice for all microsatellites to estimate the error rate.

### 2.3. Data Analyses

Mitochondrial DNA sequences were manually trimmed and the complementary sequences of each individual were assembled using Geneious R10 [49]. The sequences of each individual and those within each nest were checked for consistency. The genetic sequence obtained for each nest was compared to other publicly available sequences through BLASTn [50,51] to identify the mtDNA lineage of the locally nesting turtles to those found in other regions of the Mediterranean.

Microsatellite allele sizes were scored with Geneious R10 [49] and binned using FlexiBin v2 [52]. For each microsatellite locus at each nest, the number of alleles detected and the observed heterozygosity (H_o_) were estimated through Arlequin v3.5 [53]. Genotypes were checked for scoring errors due to stuttering, large allele dropouts, and null alleles using Micro-Checker 2.2.3 [54]. Analysis of paternity was initially checked visually by evaluating the multi-locus genotypes and the number of alleles per locus at each nest. Then, the data were analysed through the software COLONY v2.0.6.8 [37] to computationally assign sibship and parentage among individuals using likelihood methods through multi-locus genotype data with a less than 1% error rate.

## 3. Results

### 3.1. The Nests

After decades of no records of *C. caretta* nests in the Maltese islands [1], there was an unsuccessful nesting event in 2012 and a successful one in 2016 where 83.5% of eggs counted by the local authority ERA had hatched. These were followed by another successful nesting event in 2018, where all individuals hatched except one inviable egg.

The genetic results of the 120 *C. caretta* specimens presented here represent the first valuable output derived from using dead specimen samples from eight Maltese turtle nest clutches laid in the summers between 2020 and 2022. In 2020, six nests from four different beaches were identified. In four of these instances, the nest was identified when the mother laid the eggs and, thus, the nests were protected throughout the natural incubation period; meanwhile, the other two nests were not protected throughout their incubation period. The latter two were represented by the nest CRB at Ramla Bay, which is a sizable and popular sandy beach (coastline: ~400 m), and the nest CFA at Fajtata Bay, which is a small sandy beach (coastline: ~23 m) highly frequented by bathers. In 2021 and 2022, there was one recorded nesting event per year and, in 2023, two recorded nesting events (Table 1).

### 3.2. Genetic Data

In this study, a total of 858 bp of the mtDNA CR sequence was analysed. As expected, within the same clutch, all specimens exhibited the same haplotype and, overall, the clutches were represented by two haplotypes that differed from each other by 1 bp. The two haplotypes identified in this study were CC-A2.1, which represented all the studied nests, except the nest CRD, which was represented by CC-A3.1. Sequences were deposited in GenBank under accession numbers PP056536 – PP056543.

In this study, 25 microsatellite loci were analysed (Table 2), with more than 76% of the individuals producing positive scores for each locus. Analyses through Micro-Checker [54] indicated that there is no evidence for scoring errors due to stuttering, no large allele dropout, and no indication of null alleles while the genotypes of the replicates were 100% identical. Consequently, all loci were used for subsequent analyses. The number of alleles per locus varied from four alleles (Cc-2, Cc-10, Cc-17, Cc-28 and CcP5C11) to thirteen alleles in locus (CcP7D04 and Cc8E07). The mean number of alleles per locus was 7.6 ± 2.9 SD (dinucleotide loci 5.6 ± 1.6 SD; tetranucleotide loci 9.4 ± 2.7 SD). The overall heterozygosity ranged between 0.479 (Cc-2) and 0.983 (CcP7F06), with the mean heterozygosity per locus being 0.758 ± 0.144 SD (dinucleotide loci 0.701 ± 0.135 SD; tetranucleotide loci 0.810 ± 0.136 SD).

### 3.3. Sibship and Parentage Analyses

Analyses of mtDNA haplotypes allowed for the conclusion that the female that laid the eggs in the nest CRD is different from those involved in the other nests, an observation that was further confirmed through nuclear data analyses. The analyses of microsatellites through COLONY [37] indicated that the eight studied nests had originated from six mothers and seven fathers.

We found two instances where the female returned to lay the second nest within the same nesting season. The nest clutches CGA and CGB that were both laid in Għadira Bay, on 30 July 2020 and 10 August 2020, respectively, belonged to the same parents (Mother 2 and Father 2; Table 3). Therefore 11 days after laying the first clutch of 79 eggs, the mother returned to the same beach to lay a second clutch of 86 eggs. Likewise, during the same nesting season, a second female laid two nests (Mother 3 and Father 3; Table 3); the first nest having 102 eggs was laid at Ramla Bay (CRA) on 29 May 2020 and the second nest containing 92 eggs was laid at Golden Bay (CMA) on 5 July 2020. This means that Mother 3 laid two nests within 37 days, around 22 km apart.

Additionally, evident from the occurrence of more than four alleles per locus for the nestlings from the nest CRD and confirmed through COLONY [37] was the presence of multiple sires for the clutch. From the twenty-six analysed specimens, we noted that eighteen specimens (69.2%) belonged to Father 6 while eight specimens (30.8%) were fertilized by a secondary male (Father 7). We did not find evidence of polyandry in the other nests; although, some of them were represented by a few specimens, making the detection of polyandry more difficult.

## 4. Discussion

This is the first parentage study of *C. caretta* nests from Malta documenting renesting events and multiple paternity. Genetic data from the eight analysed nests indicated that the most common mtDNA CR haplotype is CC-A2.1, which is commonly proposed as the ancestral lineage for the Mediterranean Sea and was possibly introduced from the Atlantic Ocean by colonizing females in the last post-glacial period [20,21,41,55]. Currently, CC-A2.1 is the most commonly encountered haplotype, present in more than 60% of the free-living Mediterranean loggerhead sea turtles [21,56,57,58,59,60]. This haplotype dominates most Mediterranean nests and has been detected in all nesting areas studied [22,41,59,61,62]. Haplotype CC-A3.1 is the second most commonly encountered haplotype in the Mediterranean Sea and accounts for around 20% of the free-living individuals studied [21,56,57,58]. This haplotype has been recorded in nests from the eastern and southern-central Mediterranean areas, namely, Turkey, Cyprus, Lebanon, Greece and Crete, Cyprus, Libya, and Tunisia. CC-A3.1 was found to occur in high frequencies in two nesting sites, Dalyan and Dalaman in Turkey, where, in the latter, it was more common than CC-A2.1 [41]. The other haplotypes found in the Mediterranean occur at much lower frequencies in both free-living turtles and in analysed nests [41,59,60].

Nuclear data analyses showed that there were two instances where the female returned twice to a nesting beach to lay separate clutches of eggs within the same nesting season. This phenomenon is known to be common in sea turtles [63,64]. In *C. caretta*, mean renesting intervals have been reported to range between 12.7 days and 19.9 days [63]. While one female turtle returned to lay another clutch 11 days later, on the same beach, the other female turtle took much longer than reported in some other studies, laying her second clutch after 37 days and on a different beach. A comparable actual record of a similar extended renesting period of 34 days has been reported in Turkey [65]. Such delayed renesting events and the choice of a totally different nesting area may be the result of human presence and disturbance, which affect the females’ decision when opting for a nesting beach [66]. In this instance, we cannot exclude the possibility that Mother 3 laid other unrecorded nests in the interval between CMA and CRA, given that female *C. caretta* can lay more than two clutches in a season [67]. We were unable to detect cases of a female returning to nest in a different nesting season twice, up to now, since female *C. caretta* have a nesting interval of two to three years and the nests we analysed covered a shorter period of time [2,24].

In one of the nests, we were also able to detect multiple paternity resulting from polyandry. Polyandry is a common mating behaviour in sea turtles [68,69,70,71], including *C. caretta* [44,72,73,74,75,76,77]. While there are benefits to this behaviour [78], including fertilization assurance and genetic diversity benefits [79,80,81], it remains debatable whether this is a consequence of high male–female encounters. Within this scenario, low frequencies of polyandry may indicate lower chances of mating encounters, even though the sex ratios for adults in the Mediterranean are balanced [82]. In the current study, we encountered multiple paternity in 12.5% of the nesting events studied. This percentage is much lower than that noted in other Mediterranean countries, where, in Greece, more than one father was detected in nineteen out of twenty nests, with two clutches representing the contribution of at least five males [77]. A similar study in Turkey [76] revealed that multiple paternity was present in 18 out of 25 nests analysed. In the latter study, whenever multiple paternity was recorded, the majority of the offspring had a primary sire, which, on average, contributed to 62.7% of the clutch, followed by a secondary sire which, on average, contributed to 30.9%, and a tertiary sire contributing to 7.2% [76]. In our case, the primary sire contributed to 69.2% of the clutch sample analysed while the secondary sire contributed to the remaining 38.8%. Unlike some other species of turtles [83,84], *C. caretta* does not store sperm across breeding seasons [85]. Therefore the occurrence of multiple paternity is the result of multiple mating encounters and sperm storage for each breeding season, as loggerhead sea turtles rarely mate between nesting events within the season [67]. This is consistent with the observations noted in this study where the instances of renesting were represented by the same parentage. Table 3 shows that, in most instances, the mothers studied here exhibited monandry, except for the nest CRD. Consequently, the diversity of fathers noted was almost equal to that of the mothers contributing to the turtle clutches in Malta and Gozo. The genetic results of this work reveal the first details of the reproductive behaviour of *C. caretta* around the Maltese islands.

For decades, there have been no records of turtle nests from the Maltese islands [1]; although, one cannot exclude the possibility of unrecorded nests, even in some of the most human-frequented beaches, as noted in 2020. Since the first recent record in 2012, these islands experienced an increase in recorded turtle nests, with the identification of five nesting beaches, four on the island of Malta and one on the island of Gozo (Table 1; Figure 1). While increasing awareness and reporting by citizens may partially explain the increasing records of nesting events, the long absence of nesting events followed by almost yearly events between 2018 and 2023 clearly indicates an increasing trend in nesting activity around this archipelago. A trend similar to that noted in the Western Mediterranean, where, during the last decade, *C. caretta* has been expanding its nesting range and increasing nesting events in the region [6,7,86], possibly in response to global warming [7].

Impacts of human activities on nesting beaches may hinder female turtles from laying eggs or even interrupt their egg-laying activity, leading to their return later on to the same beach or to seek a quieter beach. The Maltese nests studied here were all found on beaches that are highly frequented by locals and tourists, especially during the summer months, coinciding with the peak of *C. caretta*’s nesting season between May and August [2]. Consequently, as noted in several other regions of the Mediterranean Sea, the recorded nests are highly exposed to anthropogenic impacts [7], possibly because beaches that are not frequented by humans are more difficult to monitor. Anthropogenic influence on turtle behaviour became clearer in the summer of 2020, when Malta, similar to elsewhere in the Mediterranean Sea [7], experienced a spike in nesting activities, a phenomenon that may be associated with the COVID-19 reduction in mass tourism, lowering pressures on *C. caretta* distribution and access to breeding habitats [87]. Scientific evidence shows that this species is capable of noticing changes and adjusting accordingly, moving away from the shore in the presence of disturbances [87] and reducing the number of nesting attempts in the increasing presence of artificial light [66]. While females tend to show a high variability in nest site selection [88], the presence of humans may interfere with the female turtles’ behaviour, limiting their choice. Research shows that the hatching success decreased towards the waterline; thus, nest site selection is crucial [88].

Undetected and unprotected nests are highly prone to being trampled on by beach users. One such instance was the nest CFA, in this study, which was only found after the turtles hatched, got trapped under the sand, and were found by chance. This nest was characterized by a high percentage of dead corpses of hatched individuals that found difficulty in making it through the highly compacted sand above, possibly due to sunbathers.

Knowledge of reproductive behaviour is crucial to better understand the species’ biology in an area and to be in a better position to design and manage Natura 2000 sites [39].

## 5. Conclusions

This work provides the first detailed insights into the *C. caretta* reproductive and nesting behaviour around Maltese islands through the use of genetic markers. The use of these genetic tools to understand flagship and vulnerable species in the Central Mediterranean offers an opportunity to better assess the importance of this area vis-à-vis the various behavioural aspects related to the biology of species in the region [89,90,91]. Sustained turtle research on the number of turtle egg clutches laid, nesting sites, hatching success rate, and developmental ecology are required, side by side with field research and conservation genetics research, for a complete conservation status assessment.

## Figures and Tables

**Table 1:** A list of recently recorded nesting sites in the Maltese islands. The table includes the nests’ codes and the number of dead individuals used from each respective nest during the current study.

Nesting Site	Nesting Year	Nest Reference Code in This Study	Specimens Analysed
Ġnejna Bay	2012	-	-
Golden Bay	2016	-	-
Ġnejna Bay	2018	-	-
Ramla Bay	2020	CRA	1
Ramla Bay	2020	CRB	7
Golden Bay	2020	CMA	5
Fajtata Bay	2020	CFA	23
Għadira Bay	2020	CGA	44
Għadira Bay	2020	CGB	7
Ramla Bay	2021	CRC	7
Ramla Bay	2022	CRD	26
Ramla Bay	2023	-	-
Gnejna Bay	2023	-	-

**Table 2:** The genetic data per nest, including the mtDNA haplotypes, the sample sizes per locus (n), the number of alleles identified per locus (N_a_), and the observed heterozygosity per locus (H_o_).

Nest Code	CFA	CGA	CGB	CMA	CRA	CRB	CRC	CRD	Overall
mtDNA	CC-A2.1	CC-A2.1	CC-A2.1	CC-A2.1	CC-A2.1	CC-A2.1	CC-A2.1	CC-A3.1	
cc141									
n	23	44	7	5	1	7	7	25	119
N_a_	2	3	3	2	2	3	3	4	6
H_o_	0.478	0.682	0.571	0.600	1.000	0.714	0.714	0.680	0.638
cc7									
n	23	44	7	5	1	7	7	25	119
N_a_	4	4	4	3	2	2	4	4	9
H_o_	1.000	1.000	1.000	1.000	1.000	0.714	1.000	0.720	0.924
Ccar176									
n	23	44	7	5	1	7	7	25	119
N_a_	4	2	2	3	2	2	2	2	6
H_o_	1.000	0.455	0.714	1.000	1.000	0.857	0.857	0.280	0.613
cc117									
n	22	43	7	5	1	7	7	26	118
N_a_	3	2	2	3	2	2	3	3	7
H_o_	0.955	0.535	0.286	1.000	1.000	0.571	0.857	0.577	0.653
Cc-2									
n	23	44	7	5	1	7	7	24	118
N_a_	2	2	2	1	1	2	2	4	4
H_o_	0.652	0.409	0.143	0.000	0.000	0.286	0.429	0.750	0.483
Cc-8									
n	23	44	7	5	1	7	7	26	120
N_a_	2	3	3	2	1	2	2	2	5
H_o_	0.565	0.773	0.857	0.400	0.000	0.714	0.571	0.500	0.641
Cc-10									
n	23	42	7	5	1	7	6	25	116
N_a_	3	4	4	3	2	3	3	3	4
H_o_	1.000	0.929	1.000	1.000	1.000	0.571	0.667	0.880	0.905
Cc-17									
n	23	42	7	4	1	7	6	25	115
N_a_	1	2	2	3	2	2	2	3	4
H_o_	0.000	1.000	1.000	1.000	1.000	0.571	0.333	0.400	0.609
Cc-22									
n	23	44	7	5	1	7	6	26	119
N_a_	4	2	2	2	1	4	3	3	7
H_o_	0.957	0.636	0.714	0.600	0.000	1.000	0.667	0.692	0.731
Cc-25									
n	23	42	7	5	1	7	7	26	118
N_a_	2	2	2	3	2	3	2	4	6
H_o_	0.609	0.452	0.571	0.800	1.000	1.000	0.286	1.000	0.653
Cc-28									
n	23	44	7	5	1	7	7	26	120
N_a_	3	2	2	3	2	2	2	3	4
H_o_	0.870	0.545	0.714	0.800	1.000	0.571	0.286	0.846	0.683
Cc-30									
n	22	43	7	5	1	7	7	26	118
N_a_	2	4	4	4	2	2	4	2	5
H_o_	1.000	1.000	1.000	1.000	1.000	0.143	1.000	0.692	0.881
Cc1G02									
n	23	44	7	4	1	7	7	26	119
N_a_	3	3	3	3	2	4	3	7	12
H_o_	0.696	0.727	0.857	0.750	1.000	1.000	1.000	1.000	0.824
Cc1G03									
n	23	44	7	5	1	7	7	26	120
N_a_	3	3	3	4	2	4	3	4	10
H_o_	0.696	0.705	0.714	1.000	1.000	1.000	0.429	0.884	0.758
Cc5H07									
n	23	41	7	4	1	7	5	26	114
N_a_	4	5	4	3	1	3	4	5	11
H_o_	0.957	0.976	1.000	0.500	0.000	1.000	1.000	1.000	0.956
Cc7E11									
n	22	42	7	5	1	7	6	26	116
N_a_	4	2	2	3	2	4	3	6	9
H_o_	1.000	0.452	0.429	1.000	1.000	0.857	1.000	0.923	0.741
Cc2H12									
n	23	43	7	5	1	7	6	25	117
N_a_	3	4	3	2	1	3	3	4	9
H_o_	0.957	0.627	0.857	0.400	0.000	0.857	0.333	0.680	0.701
Cc7B07									
n	23	44	7	5	1	7	7	26	120
N_a_	3	3	3	3	2	4	4	6	10
H_o_	0.957	1.000	1.000	1.000	1.000	1.000	0.714	0.962	0.967
Cc7G11									
n	23	42	7	5	1	7	5	24	114
N_a_	3	2	2	4	2	3	4	5	8
H_o_	1.000	0.452	0.571	1.000	1.000	0.857	0.800	0.708	0.692
Cc8E07									
n	22	44	7	5	1	7	7	26	119
N_a_	4	3	3	3	2	3	2	6	13
H_o_	1.000	1.000	1.000	0.800	1.000	0.571	0.571	1.000	0.941
CcP1F09									
n	23	39	7	4	1	7	6	24	111
N_a_	4	4	4	1	1	3	3	4	9
H_o_	0.652	0.949	1.000	0.000	0.000	0.857	0.500	0.958	0.820
CcP5C11									
n	23	44	7	3	1	7	7	26	118
N_a_	2	2	2	3	2	3	3	2	4
H_o_	0.609	0.500	0.429	0.667	1.000	1.000	0.571	0.500	0.559
CcP7D04									
n	23	43	7	4	1	7	6	25	116
N_a_	4	5	4	3	2	4	4	5	13
H_o_	0.913	0.953	1.000	0.500	1.000	1.000	0.667	0.960	0.922
CcP7F06									
n	23	42	7	5	1	7	6	25	116
N_a_	3	4	4	2	2	4	3	5	9
H_o_	1.000	1.000	1.000	1.000	1.000	1.000	1.000	0.920	0.983
CcP7H10									
n	23	43	7	5	1	7	7	26	119
N_a_	2	3	3	2	2	2	3	4	5
H_o_	0.522	0.721	1.000	0.600	1.000	0.571	0.857	0.577	0.664

**Table 3:** A table showing the mother and father per nest.

Nest Code	CFA	CGA	CGB	CMA	CRA	CRB	CRC	CRD
Mother	Mother 1	Mother 2	Mother 2	Mother 3	Mother 3	Mother 4	Mother 5	Mother 6
Father	Father 1	Father 2	Father 2	Father 3	Father 3	Father 4	Father 5	Father 6
								Father 7

## Data Availability

Mitochondrial DNA data related to the analyses conducted during this study are available on GenBank under accession numbers PP056536–PP056543.

## Supplementary Materials

The following supporting information can be downloaded at: https://www.mdpi.com/article/10.3390/ani14010137/s1. Table S1: List of primer, sequences (forward and reverse primers, including M13 sequence), fluorescent dye used and references.
