# Supplementary material for: Conservation Genetics of the Loggerhead Sea Turtle, Caretta caretta, from the Central Mediterranean: An Insight into the Species’ Reproductive Behaviour in Maltese Waters
Source: Animals (Basel). 2023 Dec 30;14(1):137. doi: 10.3390/ani14010137 (PMC10778046; doi:10.3390/ani14010137)
Supplement: Supplementary file 1 [file animals-14-00137-s001.zip › animals-2764933-supplementary.pdf]

Conservation Genetics of the Loggerhead Sea Turtle, *Caretta caretta*, from the Central Mediterranean: An Insight into the Species' Reproductive Behaviour in Maltese Waters

Adriana Vella, Noel Vella

Conservation Biology Research Group, Department of Biology, Faculty of Science, University of Malta
Email: adriana.vella@um.edu.mt

Supplementary material

Table S1: List of primer, sequences (forward and reverse primers, including M13 sequence), fluorescent dye used and references.

Primer		Sequence 5′ – 3′ (including M13 as used in this study)	Fluorescent dye	Reference
		**Dinucleotide loci**		
cc141	F	CAGCAGGCTGTCAGTTCTCCAC	PET	Bowen et al., 2005 [42]
	R	GGAAACAGCTATGACCATAGTACGTCTGGCCTGACTTT		
cc7	F	TGCATTGCTTGACCAATTAGTGAG	VIC	Fitzsimmons, 1998 [43]
	R	GGAAACAGCTATGACCATACATGTATAGTTGAGGAGCAAGTG		
Ccar176	F	GGCTGGGTGTCCATAAAAGA	NED	Moore and Ball, 2002 [44]
	R	GGAAACAGCTATGACCATCCCTAAGTAAAGATTGGCTGCT		
cc117	F	GGAAACAGCTATGACCATCTTTAACGTATCTCCTGTAGCTC	VIC	Martin et al., 2002 [45]
	R	CAGTAGTGTCAGTTCATTGTTTCA		
Cc-2	F	GGAAACAGCTATGACCATCCCCCATAACACCACATCTC	6-FAM	Monzón-Argüello et al., 2008 [46]
	R	AGGTCACAAATGGAGCAAGC		
Cc-8	F	GGAAACAGCTATGACCATGATGGAAACCCCTTCAAAC	PET	Monzón-Argüello et al., 2008 [46]
	R	TGTCACGGAGACACAAACATT		
Cc-10	F	TCCACATGGGGTTGTATGAA	VIC	Monzón-Argüello et al., 2008 [46]
	R	GGAAACAGCTATGACCATTGCCCTCCTTGAGAATTCAG		
Cc-17	F	CCACTGGAAGTCTAAGAAGAGTGC	NED	Monzón-Argüello et al., 2008 [46]
	R	GGAAACAGCTATGACCATGGAATTGAAGGGATTTTGCT		
Cc-22	F	GGAAACAGCTATGACCATCCCCCACTGCTTTAACTTCA	VIC	Monzón-Argüello et al., 2008 [46]
	R	TATTCCAACATGCCCACAGA		
Cc-25	F	GGAAACAGCTATGACCATTTTGCTTTCCCCATCTGAC	VIC	Monzón-Argüello et al., 2008 [46]
	R	AGCCTCCAGCACAGCATTAT		
Cc-28	F	AGCCCATATGTTTCCCTTCA	NED	Monzón-Argüello et al., 2008 [46]
	R	GGAAACAGCTATGACCATTGGCCCATCTTATTTCAGTG		
Cc-30	F	CTTTTGGAGGCAGGCTAGTG	6-FAM	Monzón-Argüello et al., 2008 [46]
	R	GGAAACAGCTATGACCATGAAGCCAGGTTGATCAGGAG		
		**Tetranucleotide loci**		
Cc1G02	F	GGAAACAGCTATGACCATAGGTGCCTAAACATTGATAGT	6-FAM	Shamblin et al., 2007 [47]
	R	GTTTATACTGTGCTCTTTCGTGTAAT		
Cc1G03	F	GGAAACAGCTATGACCATGGAATGTGCAGAATGTATGT	NED	Shamblin et al., 2007 [47]
	R	GTTTAACGAGCATGTATCTAAAGTAA		

Cc5H07	F	GGAAACAGCTATGACCATAAGGAAGGAGAGGCTTATTAC	NED	Shamblin et al., 2007 [47]
	R	GTTTGGGCAATGAGACTGGAACTA		
Cc7E11	F	GTTTGAAGAGCTGACCCCATATAG	PET	Shamblin et al., 2007 [47]
	R	GGAAACAGCTATGACCATAAACACAGAAATGAGGGATAG		
Cc2H12	F	GGAAACAGCTATGACCATTCTTCAGGAGTTTTTGACTTG	6-FAM	Shamblin et al., 2007 [47]
	R	GTTTCCACACCCCTGTTTCAGA		
Cc7B07	F	GTTTATAATGTTGGTGAGCAATATAG	NED	Shamblin et al., 2007 [47]
	R	GGAAACAGCTATGACCATCAGGAGTTAAACCAGGCACAGT		
Cc7G11	F	GTTTCTCAGCATGAAAGTGTAATAC	NED	Shamblin et al., 2007 [47]
	R	GGAAACAGCTATGACCATGGATTTGTTTCAGTAATAG		
Cc8E07	F	GTTTAGCACTGGGTTGTGTGATTA	6-FAM	Shamblin et al., 2007 [47]
	R	GGAAACAGCTATGACCATACCACCATTCTGATTTGTTAG		
CcP1F09	F	GGAAACAGCTATGACCATAAATGTTGCCGTTTCTATTG	6-FAM	Shamblin et al., 2009 [48]
	R	GTTTCTCCCCCAACCTCTCAT		
CcP5C11	F	GTTTCTATTTGACACCACTCCACTCT	PET	Shamblin et al., 2009 [48]
	R	GGAAACAGCTATGACCATTTGATTCTCTTCCCTTTACA		
CcP7D04	F	GGAAACAGCTATGACCATGAGCAAAGTAACCCTAACA	NED	Shamblin et al., 2009 [48]
	R	GTTTGGAGCCAAATTAGAGATCAAC		
CcP7F06	F	GGAAACAGCTATGACCATGACCACCCTCCAGTGAA	VIC	Shamblin et al., 2009 [48]
	R	GTTTGTGTTGGTTGATTTACTTCTATG		
CcP7H10	F	GGAAACAGCTATGACCATCGAGACTCAATAAATACAGAGA	PET	Shamblin et al., 2009 [48]
	R	GTTTATAACAACACAGCATAAAATAAT		

Microsatellite amplification protocols used followed Monzón-Argüello et al. [46] and Shamblin et al. [47,48].

References:

42. Bowen, B.W.; Bass, A.L.; Soares, L.; Toonen, R.J. Conservation implications of complex population structure: Lessons from the loggerhead turtle (*Caretta caretta*). *Mol. Ecol.* **2005**, *14*, 2389–2402, doi:10.1111/j.1365-294X.2005.02598.x.

43. Fitzsimmons, N.N. Single paternity of clutches and sperm storage in the promiscuous green turtle (*Chelonia mydas*). *Mol. Ecol.* **1998**, *7*, 575–584, doi:10.1046/j.1365-294x.1998.00355.x.

44. Moore, M.K.; Ball, R.M. Multiple paternity in loggerhead turtle (*Caretta caretta*) nests on Melbourne Beach, Florida: A microsatellite analysis. *Mol. Ecol.* **2002**, *11*, 281–288, doi:10.1046/j.1365-294X.2002.01426.x.

45. Martin, A.P.; Pardini, A.T.; Noble, L.R.; Jones, C.S. Conservation of a dinucleotide simple sequence repeat locus in sharks. *Mol. Phylogenet. Evol.* **2002**, *23*, 205–213, doi:10.1016/S1055-7903(02)00001-5.

46. Monzón-Argüello, C.; Muñoz, J.; Marco, A.; López-Jurado, L.F.; Rico, C. Twelve new polymorphic microsatellite markers from the loggerhead sea turtle (*Caretta caretta*) and cross-species amplification on other marine turtle species. *Conserv. Genet.* **2008**, *9*, 1045–1049, doi:10.1007/s10592-007-9446-4.

47. Shamblin, B.M.; Faircloth, B.C.; Dodd, M.; Wood-Jones, A.; Castleberry, S.B.; Carroll, J.P.; Nairn, C.J. Tetranucleotide microsatellites from the loggerhead sea turtle (*Caretta caretta*). *Mol. Ecol. Notes*

2007, *7*, 784–787, doi:10.1111/j.1471-8286.2007.01701.x.

48. Shamblin, B.M.; Faircloth, B.C.; Dodd, M.G.; Bagley, D.A.; Ehrhart, L.M.; Dutton, P.H.; Frey, A.; Nairn, C.J. Tetranucleotide markers from the loggerhead sea turtle (*Caretta caretta*) and their cross-amplification in other marine turtle species. *Conserv. Genet.* **2009**, *10*, 577–580, doi:10.1007/s10592-008-9573-6.
